# Arachidonic acid reverses cholesterol and zinc inhibition of human voltage-gated proton channels

**DOI:** 10.1016/j.jbc.2023.104918

**Published:** 2023-06-12

**Authors:** Shuo Han, Sarah Applewhite, Jenna DeCata, Samuel Jones, John Cummings, Shizhen Wang

**Affiliations:** Division of Biological and Biomedical Systems, School of Science and Engineering, University of Missouri-Kansas City, Kansas City, Missouri, USA

**Keywords:** voltage-gated proton channel, voltage sensor, arachidonic acid, ligand gating, conformational dynamics, single-molecule FRET

## Abstract

Unlike other members of the voltage-gated ion channel superfamily, voltage-gated proton (Hv) channels are solely composed of voltage sensor domains without separate ion-conducting pores. Due to their unique dependence on both voltage and transmembrane pH gradients, Hv channels normally open to mediate proton efflux. Multiple cellular ligands were also found to regulate the function of Hv channels, including Zn^2+^, cholesterol, polyunsaturated arachidonic acid, and albumin. Our previous work showed that Zn^2+^ and cholesterol inhibit the human voltage-gated proton channel (hHv1) by stabilizing its S4 segment at resting state conformations. Released from phospholipids by phospholipase A2 in cells upon infection or injury, arachidonic acid regulates the function of many ion channels, including hHv1. In the present work, we examined the effects of arachidonic acid on purified hHv1 channels using liposome flux assays and revealed underlying structural mechanisms using single-molecule FRET. Our data indicated that arachidonic acid strongly activates hHv1 channels by promoting transitions of the S4 segment toward opening or “preopening” conformations. Moreover, we found that arachidonic acid even activates hHv1 channels inhibited by Zn^2+^ and cholesterol, providing a biophysical mechanism to activate hHv1 channels in nonexcitable cells upon infection or injury.

Voltage-gated proton (Hv) channels are expressed in many cell types in the human body, such as phagocytes, glial cells, cardiomyocytes, pancreatic islet β-cells, and sperm (1). In phagocytes, Hv channels compensate charge and pH imbalances caused by NADPH oxidases, thus facilitating respiratory bursts to eliminate invasive pathogens (2, 3); in cardiomyocytes, Hv channels are important for pH homeostasis by providing an effective acid disposal mechanism without Na^+^ overload through Na^+^/H^+^ or Na^+^/HCO3^-^ pathways (4); in glial cells, Hv channels exacerbate ischemic damage by facilitating NADPH oxidases to produce reactive oxygen species (5); in human sperm, acid extrusion through hHv1 channels mediate alkalinization crucial for capacitation and hyperactivation (6, 7, 8). In addition, hHv1 channels are highly expressed in invasive breast cancer cells and malignant B cells (9, 10).

Unlike canonical voltage-gated ion channels (11), Hv channels only contain voltage sensors without separate ion conductive pore domains (12, 13). As bona fide stand-alone voltage sensors, the transmembrane domain of hHv1 channels consists of four transmembrane segments, named S1 through S4, with the S4 segment carrying multiple positively charged residues driven outward by depolarization voltages to activate proton conductive pathway (13). Hv channels conduct protons with almost perfect selectivity, and their voltage dependences are remarkably shifted by transmembrane pH (proton) gradients as high as 40 mV per unit (14, 15). As a result, Hv channels normally open when the proton electrochemical gradient is directed outward, thus serving as acid extruders (1). In addition to voltage and pH, Hv channels are regulated by many ligands, including Zn^2+^ (16, 17), cholesterol (18), albumin (19), polyunsaturated arachidonic acid (20) and ATP (21), which may also play important physiological roles in cells. Mechanisms of Zn^2+^ and cholesterol inhibition in hHv1 channels have been studied extensively (18, 22, 23), but arachidonic acid activation offers an opportunity to understand the ligand activation mechanism in Hv channels. In many nonexcitable cells like phagocytes, their membrane cholesterol levels normally do not change dramatically, and in fact, there was evidence suggesting that Hv channels are located within cholesterol-enriched lipid raft domains (24, 25, 26). Since Hv channels play important roles in maintaining cellular pH homeostasis (2, 3), how do they overcome cholesterol inhibition to mediate proton efflux under conditions like respiration burst? We hypothesized that arachidonic acid might play a critical role, for it is also a strong activator for NADPH oxidases (27, 28).

In the present work, we performed functional characterization of purified human voltage-gated proton channel hHv1 reconstituted into liposomes using fluorescence liposome flux assays, which allowed us to control cholesterol and arachidonic acid levels precisely. Our data indicated that arachidonic acid strongly activates hHv1 channels, even those inhibited by cholesterol and Zn^2+^. With single-molecule FRET (smFRET) measurements, we further showed that arachidonic acid modified the conformational dynamics of the S4 segment, a mechanism also utilized by other gating factors, including voltage, pH, cholesterol, and Zn^2+^, to regulate its channel function (18, 29). Our results suggest that the release of arachidonic acid may serve as a key mechanism to activate hHv1 channels in cells under infection, injury, or inflammation status.

## Results

### Arachidonic acid activates hHv1 channels reconstituted into liposomes

**Figure 1:**
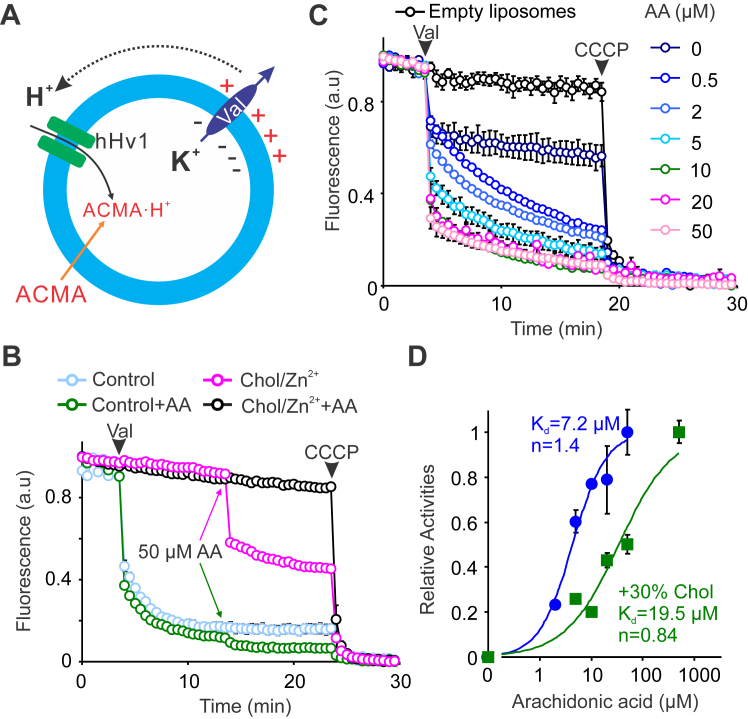
**Arachidonic acid activates hHv1 channels and reverses the Zn**^**2+**^**and cholesterol inhibition**. *A*, liposome flux assay to determine proton uptake through purified hHv1 channels reconstituted into liposomes. Driven by electrical potential generated by the K^+^ gradient across liposomes in the presence of 0.45 μM valinomycin, proton uptake into liposomes is reported by quenching of ACMA fluorescence due to protonation. *B*, arachidonic acid reversed the inhibition of hHv1 channels by 30% cholesterol and 0.5 mM Zn^2+^. Zn^2+^ was added to both sides of liposomes, and flux assays were started by adding 0.45 μM K^+^ ionophore valinomycin at 4 min time point (*black arrow*), then 50 μM arachidonic acid (AA) was added at the 14 min time point, as indicated by *blue* and *green arrows*. All data were presented as mean ± s.e, n = 3. *C*, arachidonic acid activates hHv1 channels in a dose-dependent manner. The protein/lipid ratio was decreased to 1/10,000 (w/w), so arachidonic acid activation can be quantified. All data were presented as mean ± s.e, n = 3. *D*, arachidonic acid activation of hHv1 channels in the presence or absence of 30% cholesterol. Proton uptake activities of hHv1 channels were normalized between 0 and 1. Relative activities of hHv1 channels at different concentrations of arachidonic acid were fitted with the Hill equation to yield apparent half activation concentration (Kd) and Hill coefficient, and all data were presented as mean ± s.e, n = 3. ACMA, 9-amino-6-chloro-2-methoxyacridine.

In addition to voltage and pH (14), many cellular ligands were also reported to regulate hHv1 channels, including inhibitors like Zn^2+^ (17) and membrane cholesterol (18) and activators like arachidonic acid (20), albumin (19), and ATP (21). Although patch-clamp studies have characterized the effects of these ligands on hHv1 channels individually, the function of hHv1 channels in the presence of multiple cellular ligands remained unclear. In the present work, we examined the function of purified hHv1 proteins reconstituted into liposomes by liposome flux assays to gain mechanistic insights into ligand gating. With reconstituted liposome samples, concentrations of arachidonic acid and membrane cholesterol were controlled precisely. Liposome flux assay measures the quenching of pH-sensitive fluorophore 9-amino-6-chloro-2-methoxyacridine (ACMA) due to proton uptake into liposomes through hHv1 channels, driven by transliposomal K^+^ electrical potentials (Fig. 1*A*). In liposomes containing 30% cholesterol (w/w) with 0.5 mM Zn^2+^ on both sides, hHv1 channels were completely inhibited, which was remarkably reversed by 50 μM arachidonic acid (Fig. 1*B*). Without cholesterol and Zn^2+^, 50 μM arachidonic acid only slightly increased the rate of ACMA quenching. We reasoned that proton uptake into liposomes *via* hHv1 channels perhaps reaches saturation too quickly to reflect the robust activation effects of arachidonic acid. We then lowered the protein/lipid ratio of reconstitution from 1:200 to 1:10,000 (w/w), which decreased the number of hHv1 proteins in each liposome, therefore slowing down the rate of proton uptake into liposomes, as shown previously by Lee *et al*. (30). Indeed, with the low protein–lipid ratio liposomes, the rate of proton uptake was significantly slower, which was promoted by arachidonic acid significantly in a dose-dependent manner (Fig. 1*C*). We fitted the relative proton uptake activities *versus* arachidonic acid concentration curve with the Hill equation, which yielded a half activation concentration of 7.2 μM (Fig. 1*D*). Membrane cholesterol levels of most mammalian cells are not changed dramatically, except for in specialized cells like sperm during the capacitation process (31). With hHv1 liposomes containing 30% cholesterol, we examined the effects of arachidonic acid on the proton uptake, which, as shown in Figure 1*D*, demonstrated a significant shift in half activation concentration by ∼3 fold to 19.5 μM. Interestingly, the latter is very close to that obtained from electrophysiological studies performed on Hv1 channels expressed in HEK293 cells (20). Our functional studies unambiguously indicated that arachidonic acid can attenuate or even reverse cholesterol and Zn^2+^ inhibition, which may serve as an important mechanism to activate hHv1 channels in cells upon infection or injury.

### Arachidonic acid stabilizes the S4 segment at opening conformations

**Figure 2:**
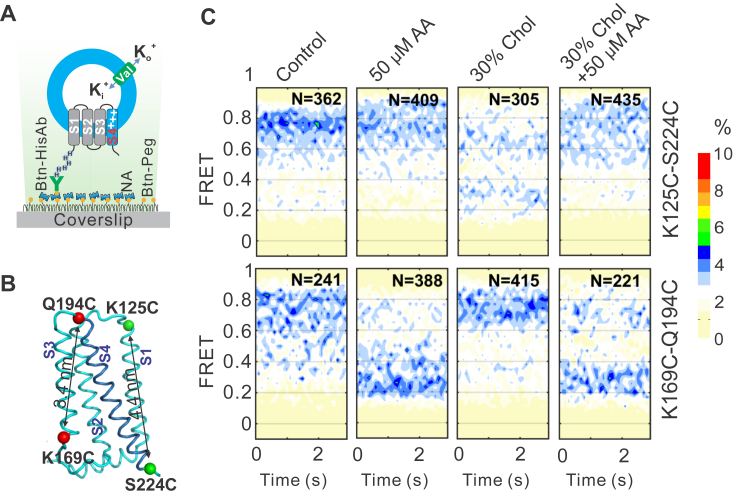
**Arachidonic acid stabilizes the S4 segment at intermediate/opening conformations**. *A*, the experimental setup to examine conformational transitions of the hHv1 S4 segment by smFRET. The PEG passivated coverslip surface contained 2% biotin-PEG (Btn-Peg), which retained biotinylated anti-His tag antibodies (Btn-His Ab) through neutravidin (NA). The biotinylated anti-His tag antibodies immobilized hHv1 proteoliposomes with N-terminal 6xHis tag facing outside for smFRET imaging. *B*, ribbon diagram hHv1 channel (PDB#5OQK) structure with the S4 segment color *purple*. Residues of two labeling sites, K125-S224 and K169-Q194, were highlighted as *green* and *red spheres*, respectively. *C*, FRET contour maps of smFRET data collected from the K125C-S224C (*upper panels*) and K169C-Q194C (*lower panels*) labeling sites under −85 mV. The data from the first 3 s of all smFRET traces were used to generate FRET contour maps with a bin size of 0.03 and trace number presented as N in each panel. PDB, Protein Data Bank; smFRET, single-molecule fluorescence resonance energy transfer.

Our previous studies have indicated that cholesterol and Zn^2+^ inhibit hHv1 channels by stabilizing the S4 segment in the hHv1 voltage sensor at resting state conformations (18). Since arachidonic acid can reverse cholesterol and Zn^2+^ inhibition, we hypothesized that arachidonic acid may also act on the S4 segment to activate the hHv1 channel. We previously showed that the S4 segment in the hHv1 voltage sensor exhibited spontaneous transitions among three major conformational states, as reflected by smFRET measurements collected on two hHv1 mutants with FRET fluorophore pair conjugated to either K125C-S224C or K169C-Q194C sites (18, 29). We showed that activating voltages enriched the high FRET population when donor/acceptor fluorophores were labeled at the K125C-S224C labeling sites but the low FRET population at the K169C-Q194C labeling sites. FRET changes at the two different labeling sites consistently suggested that the S4 segment was driven toward the extracellular side upon activation (29). In the present work, we first confirmed that arachidonic acid also reversed cholesterol and zinc inhibition of two hHv1 mutants for smFRET studies (Fig. S1). Using smFRET, we further examined the effects of 50 μM arachidonic acid in the absence or presence of 30% cholesterol on the S4 conformational dynamics. FRET contour maps obtained at the two labeling sites did not change over 3 s, indicating that smFRET data were collected at equilibrium states (Fig. 2*C*). In the presence of 50 μM arachidonic acid, 30% cholesterol, or both, raw FRET histograms calculated from all smFRET traces at the two labeling sites indicated that the conformational (FRET) distributions of the S4 segments were much less voltage-dependent, in sharp contrast to those collected from the control condition (Fig. S2). This may be explained by the fact that arachidonic acid reduced the portion of hHv1 channels available for voltage activation, while cholesterol suppressed them from being activated by voltage. Therefore, we only focused on the smFRET data collected under the resting voltage of −85 mV. At the K125C-S224C labeling sites, FRET contour maps showed that 50 μM arachidonic acid significantly shifted the distributions toward high FRET populations and was able to reverse the effects of 30% cholesterol (Fig. 2*C*), while FRET data from K169C-Q194C labeling sites showed that arachidonic acid enriched lower FRET populations and also reversed the effects of 30% cholesterol (Fig. 2*C*). FRET changes induced by 50 μM arachidonic acid collected from the two labeling sites consistently indicated that arachidonic acid stabilized the S4 segment at the extracellular side to activate hHv1 channels. Moreover, our smFRET data revealed that the arachidonic acid reversed the cholesterol inhibition by eliminating its conformational effects on the hHv1 channel, that is, stabilizing the S4 segment at inward resting conformations.

### Arachidonic acid modifies the dynamics of the S4 segment

**Figure 3:**
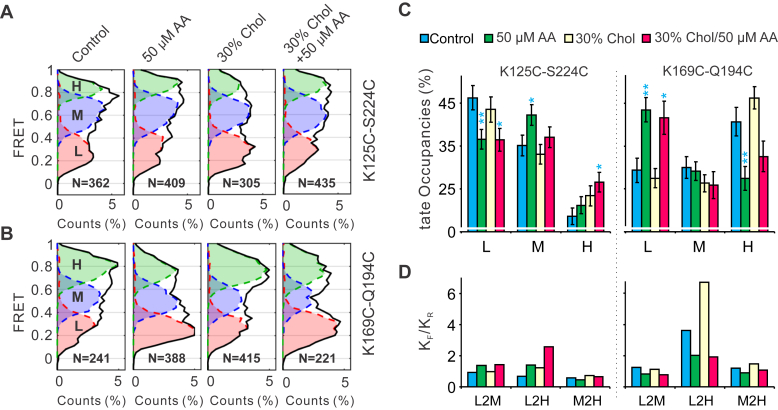
**Effects of arachidonic acid on conformational kinetics of the hHv1 S4 segment**. *A* and *B*, FRET histograms from K125C-S224C and K169C-Q194C labeling sites, collected under −85 mV, in the absence or presence of either 50 μM arachidonic acid, 30% (w/w) cholesterol, or both. FRET histograms contained low (L, *red*), medium (M, *purple*), and high (H, *green*) FRET populations. *C*, FRET state occupancies of low (L), medium (M), and high (H) FRET states at K125C-S224C and K169C-Q194C labeling sites. The numbers of traces are 362, 409, 305, and 435 at K125C-S224C sites and 241, 388, 415, and 221 at K169C-Q194C sites, respectively. Student’s *t* tests were performed to examine the significance levels of FRET state occupancies between the control and ligand groups. The *p*-values were calculated from the FRET state occupancy, s.e., and trace number N of the control and ligand groups, using the *t* test calculator of GraphPad (Dotmatics Inc). The probability (p) of the null hypothesis (*i.e.*, FRET state occupancies between the control and ligand groups have no significant difference) was indicated as ∗ for *p* < 0.05 or ∗∗ for *p* < 0.01. *D*, equilibrium constants (K_EQ_) of FRET transitions, calculated as ratios of forward (K_F_) to reverse (K_R_) transition rates between low and medium (L2M), low and high (L2H), and medium and high (M2H) FRET states.

To further quantify the changes in conformational distributions of the S4 segment, we performed kinetic analyses on smFRET traces collected from the two labeling sites using a kinetic model containing three FRET states developed previously (29). The smFRET traces from K125C-S224C labeling sites were idealized into low, medium, and high FRET states with peak centers at 0.27, 0.6, and 0.9 and those from K169C-Q194C sites at 0.24, 0.52, and 0.78, respectively (Figs. 3, *A* and *B* and S3). The FRET state occupancy data indicated that arachidonic acid promoted the FRET-0.6 state and decreased the FRET-0.27 states at the K125C-S224C labeling sites (Fig. 3*C*). Corresponding changes were observed at K169C-Q194C labeling sites, where arachidonic acid remarkably promoted the low FRET-0.24 and suppressed the high FRET-0.78 states (Fig. 3*C*). The state occupancy data from the two labeling sites supported that arachidonic acid stabilized the S4 segment at conformations that hHv1 channels were at either activated or “pre-activated” states.

Since our smFRET data were collected under an equilibrium state, the equilibrium constants (K_EQ_) of different FRET transitions can be calculated as ratios of rates between the forward (K_F_) and backward (K_R_) transitions. As shown in Figure 3*D*, activatory ligand arachidonic acid and inhibitory cholesterol have distinct effects on the equilibrium constants of low to medium (L2M) and low to high (L2H) FRET states. At the K125C-S224C labeling sites, arachidonic acid shifts the K_EQ_ of the L2M transition toward the medium FRET states and the L2H transition toward the high FRET state. At K169C-Q194C labeling sites, arachidonic acid shifts the L2M and L2H transitions toward the lower FRET states. Shifts in K_EQ_ of different transition types by arachidonic acid matched its effects in modifying conformational landscapes of the S4 segment in the hHv1 channel, indicating that it promotes channel openings by stabilizing the S4 segment at extracellular sides.

## Discussion

**Figure 4:**
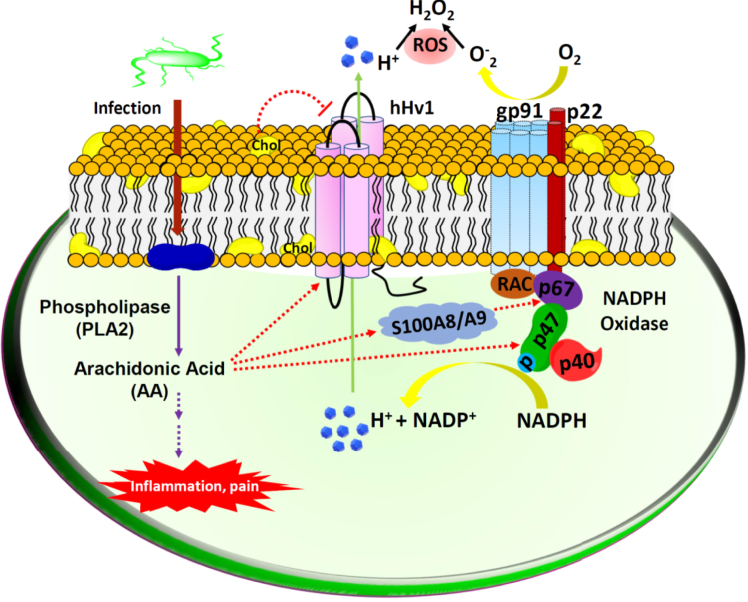
**The role of arachidonic acid in activating hHv1 and NADPH oxidases to facilitate respiration bursts in phagocytes**. Upon injury or infection, activation of phospholipase A2 releases arachidonic acid, the precursor molecule of many inflammatory mediators. Arachidonic acid activates NADPH oxidases directly through p47 (27) and indirectly through the S100A8/A9 complex, which interacts with p67/RAC-2 (28). Membrane cholesterol (Chol) inhibits hHv1 channels, while arachidonic acid directly activates hHv1 channels to extrude protons released by NADPH oxidases to facilitate the production of reactive oxygen species (ROS).

Polyunsaturated fatty acid, arachidonic acid, is the precursor of many proinflammatory lipid molecules and directly regulates the function of many ion channels (32). In the present work, with purified hHv1 channels reconstituted into liposomes, our liposome flux assays further confirmed the robust activatory effects of arachidonic acid, which increased both rates and the steady state of the ACMA quenching (Fig. 1, *B* and *C*). In our liposome flux assays, the K^+^ gradient generated a voltage of ∼60 mV across liposomes with a negative interior. If hHv1 channels adopted random orientations, hHv1 channels with their cytoplasmic domain at the intraliposomal side were exposed to a voltage of −60 mV, lower than its threshold activating voltage (33). With a very low protein–lipid ratio of 1/10,000 (w/w), most liposomes contained only one hHv1 channel protein, with ∼50% of liposomes carrying hHv1 channels with their cytoplasmic domains at the intraliposomal side (29, 30). As a result, steady-state quenching of ACMA fluorescence only reached about 50%, in sharp contrast to ∼90% in liposomes containing 50× hHv1 proteins with both orientations (Fig. 1, *B* and *C*). Our liposome flux data indicated that arachidonic acid almost doubled the steady state ACMA quenching, implying that it perhaps shifts the threshold activating voltage of the hHv1 channel under −60 mV (20), so liposomes carrying hHv1 channels with either orientation both contributed to uptake protons (Fig. 1*C*). More importantly, we showed that arachidonic acid was able to activate hHv1 channels inhibited by both 30% (w/w) cholesterol and 0.5 mM Zn^2+^, while cholesterol contents in most cell membranes are about 10 to 30% and Zn^2+^ in the human blood plasma is only ∼20 μM (34, 35). In nonexcitable cells like macrophages, membrane cholesterol and extracellular Zn^2+^ probably do not undergo dramatic changes. When cells are injured or infected, hHv1 channels, as acid extruders due to their unique voltage/pH dependencies (1), are inhibited by membrane cholesterol and Zn^2+^. However, releasing arachidonic acid could serve as a key mechanism to activate hHv1 channels. Intriguingly, arachidonic acid also stimulates NADPH oxidases in phagocytes, which require hHv1 channels to extrude protons released during respiration bursts (27, 28, 36). As shown in Figure 4, our results suggest that arachidonic acid release through the phospholipase A2 signaling pathway may be an important mechanism to activate hHv1 channels essential for respiration burst in cells upon infection or injury (37, 38).

Our previous studies showed that inhibitors of the hHv1 channels, including Zn^2+^ and cholesterol, stabilize the S4 segment at resting state conformations to suppress channel openings (18, 29). Identification of arachidonic acid as a strong activator of the hHv1 channel allowed us to understand the mechanisms underlying ligand activation. In the present work, our smFRET studies suggested that arachidonic acid also significantly impacts the S4 dynamics by enriching conformations representing intermediate and activating states, even under a strong resting voltage of −85 mV (Figs. 2*C* and 3*C*). Our results further established the S4 segment as the central gating machinery of the hHv1 channel. In the presence of 50 μM arachidonic acid, the voltage dependences of conformational distributions of the S4 segment, observed at both the K125C-S224C and K169C-Q194C sites, were significantly diminished, in sharp contrast to those under control conditions (Fig. S2). These data suggested that hHv1 channels under −85 mV may be promoted to activated states by 50 μM arachidonic acid, supporting our liposome flux results, showing that arachidonic acid decreased steady-state quenching of ACMA fluorescence (Fig. 1*C*). However, such a dramatic shift in activating voltage was not observed on the hHv1 channels expressed in mammalian cells (20), perhaps due to the presence of membrane cholesterol. Our liposome assays also showed that 50 μM arachidonic acid did not fully reverse cholesterol inhibition by promoting ACMA quenching to ∼90%, compared to those without cholesterol (Fig. 1, *B* and *C*). In addition, we also uncovered that arachidonic acid reversed the conformational effects of cholesterol on the S4 segment as the underlying structural basis to reverse cholesterol inhibition. Our previous study showed that cholesterol effects were very specific, and many cholesterol analogs, like desmosterol and hydroxycholesterol, do not inhibit hHv1 channels (18). Similar observations were made on mouse Hv1 channels using arachidonic acid, which showed that both the unsaturated acyl chain and the carboxyl group are required for its activation effects (20). Although these results proposed that cholesterol and arachidonic acid may regulate hHv1 channels through direct binding, no conclusive evidence is yet available to rule out their effects by impacting the physical properties of membranes. In future work, binding sites of cholesterol and arachidonic acid need to be determined using cryo-EM or mutagenesis analysis to confirm their direct interactions with hHv1 channels, a key knowledge which is also important to understand their physiological or pathophysiological roles in different cells like macrophages, microglia, and cancer cells.

## Experimental procedures

### Protein expression and purification

The codon-optimized hHv1 complementary DNA was synthesized (GenScript Inc) and subcloned into pET-28a between the NdeI and BamHI sites. Mutations were introduced into the hHv1 channel by site-directed mutagenesis kit (Agilent Inc) and confirmed by DNA sequencing. As described previously (18, 29), all hHv1 mutants were overexpressed in *Escherichia coli* BL21(DE3) cells; purification of the hHv1 proteins carrying N-terminal 6∗histidine fusion tag was performed by metal affinity chromatography; all hHv1 mutant proteins for smFRET studies carried the N214R mutation (29) and were conjugated with Cy3/Cy5 maleimide *via* introduced cysteine residues.

### Liposome reconstitution and flux assay

For smFRET studies, hHv1 proteins labeled with Cy3/Cy5 fluorophore pair were mixed with 1-palmitoyl-2-oleoyl-sn-glycero-3-phosphoethanolamine/1-palmitoyl-2-oleoyl-sn-glycero-3-phospho-(1′-rac-glycerol) (3/1, w/w) lipids dissolved in 10 mM Fos-Choline 12 at a protein/lipid ratio of 1/4000 (w/w), and hHv1 proteoliposomes were formed by removing the detergent Fos-Choline 12 by dialysis overnight under 4 °C against the reconstitution buffer containing 20 mM Hepes, 150 mM KCl, 0.05 mM NaCl, 1 mM Tris(2-carboxyethyl)phosphine hydrochloride, pH 7.5. For liposome flux assays, hHv1 proteins were mixed with 1-palmitoyl-2-oleoyl-sn-glycero-3-phosphoethanolamine/1-palmitoyl-2-oleoyl-sn-glycero-3-phospho-(1′-rac-glycerol) (3/1, w/w) lipids at ratios of either 1/200 or 1/10,000 (w/w), and hHv1 proteoliposomes were formed by detergent removal using Bio-Beads SM-2 (Bio-rad Inc) for overnight under 4 °C.

Liposome flux assays were performed by adding 20 μl of hHv1 proteoliposomes into 180 μl flux assay buffer containing 20 mM Hepes, 150 mM N-methyl-D-glucamine, 0.2 μM pH-sensitive fluorophore ACMA, pH 7.5, creating a transliposomal K^+^ gradient of 10 fold (150 mM inside *versus* 15 mM outside). The liposome flux assays were triggered by adding valinomycin at a final concentration of 0.45 μM, and ACMA fluorescence intensities were monitored by a 96-well plate reader (Ex/Em = 390/460 nm). Proton-specific ionophore carbonyl cyanide m-chlorophenyl hydrazone was used as a positive control. The proton uptake activities of hHv1 channels were calculated as rates of ACMA quenching after adding valinomycin, as described (39). To test the effects of arachidonic acid, hHv1 channels were reconstituted into liposomes with 30% cholesterol (w/w), and arachidonic acid stock (5 mM dissolved in ethanol) was added during liposome flux assays to reach different final concentrations. For hHv1 liposomes without cholesterol, arachidonic acid was added at different concentrations before liposome flux assays started. Proteoliposomes containing hHv1 WT proteins were included in every batch of liposome flux assays for normalization.

### smFRET imaging and kinetic analysis

All smFRET measurements were performed on a customized objective-based total internal reflection fluorescence (TIRF) microscope equipped with Apo-TIRF NA1.49 objective lens (Nikon Inc), Cobolt 532/640 nm laser lines (HÜbner Photonics Inc), ImagEM X2 camera, and W-View GEMINI beam splitter (Hamamatsu Inc) containing the ET-532/640 nm laser dual-band filter set (Chroma Inc). Sample chambers with PEG passivated surfaces for smFRET imaging were prepared as described (40). The hHv1 proteoliposomes were immobilized on the coverslip surfaces by biotinylated anti-His tag antibodies (Invitrogen#MA1-21315-BTIN) attached to the biotinylated coverslip surface through neutravidin as shown in Figure 2*A*. Donor fluorophores were excited by a 532 nm laser (∼1 W/cm^2^), and time-lapse movies were collected by HCImage Live software (Hamamatsu Inc, https://hcimage.com/hcimage-overview/hcimage-live/) with an exposure time of 100 msec per frame. The typical recording time is ∼3 min or when over 95% of donor or acceptor fluorophores bleached. All imaging buffers contained ∼3 mM Trolox, 5 mM protocatechuic acid, and ∼15 μg/μL of protocatechuate-3,4-dioxygenase to remove the oxygen, thus enhancing the photostabilities of fluorophores. For every mutant/voltage/ligand condition, at least three independent batches of smFRET movies were collected. The raw movies were converted into.tiff stacks without any correction or adjustment, and raw smFRET traces were identified and extracted by the SPARTAN software developed by the Blanchard group (https://www.scottcblanchardlab.com/software), using a point spread function size of seven pixels (41). All raw smFRET traces passed automatic selection criteria (FRET lifetime > 5 s; donor/acceptor correlation coefficient between −1.1 and 0.5; signal/noise >8; background noise <70; Cy3 blinks <4; overlap molecules removed) and were further visually inspected by two researchers independently following the criteria described previously (42).

All smFRET traces passed the SPARTAN software automatic selections, and manual inspections were included in kinetic analyses. FRET contour maps were calculated from smFRET trace data of the first 3 s, and FRET histograms were calculated from all data points of all traces, with every data point contributing equally and bin size as 0.03. All smFRET traces were idealized based on a kinetic model containing four FRET states, including low (L), medium (M), and high (H) FRET states, with one additional blinking state (B) to minimize the impacts of blinking or bleaching events (29). Based on our previous results, the peak centers for low, medium, and high FRET states at K125C-S224C sites were fixed at 0.27, 0.6, and 0.9 and at K169C-Q194C sites at 0.24, 0.52, and 0.78, respectively (29). The maximum point likelihood algorithm (43) in SPARTAN software was used to optimize rate constants of smFRET datasets collected from different labeling sites, under voltages and ligand conditions (41).

## Data availability

All data are contained within the manuscript.

## Supporting information

This article contains supporting information.

## Conflict of interest

The authors declare that they have no conflicts of interest with the contents of this article.
