# Supplementary material for: Arachidonic acid reverses cholesterol and zinc inhibition of human voltage-gated proton channels
Source: J Biol Chem. 2023 Jun 12;299(7):104918. doi: 10.1016/j.jbc.2023.104918 (PMC10344949; doi:10.1016/j.jbc.2023.104918)
Supplement: Supporting information [file mmc1.pdf]

Supplementary Materials

Arachidonic acid reverses cholesterol and zinc inhibition of human voltage-gated proton channels

Shuo Han[1], Sarah Applewhite[1], Jenna DeCata[1], Samuel Jones[1],

John Cummings[1] and Shizhen Wang[1*]

[1]Division of Biological and Biomedical Systems

School of Science and Engineering

University of Missouri-Kansas City, Kansas City, MO 64110 USA

*Corresponding to SW (wangshizhen@umkc.edu)

Running title: Ligand activation of the hHv1 channel

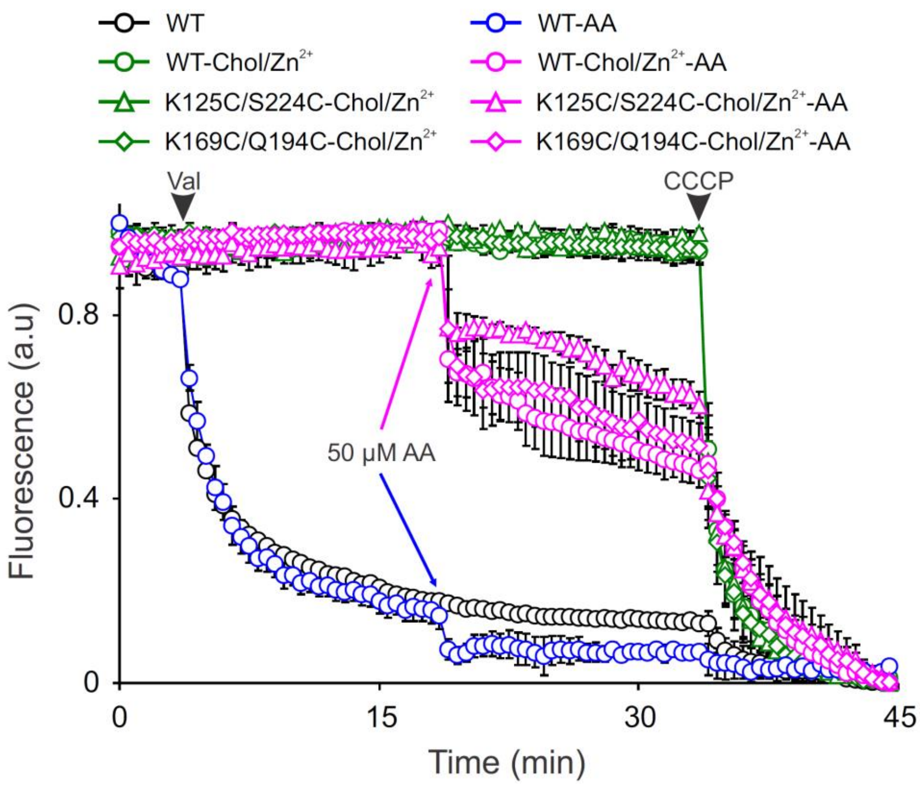

Fig S1. Arachidonic acid reversed cholesterol and zinc inhibition of 2 hHv1 mutants for smFRET studies.

The 2 hHv1 mutants for smFRET studies, including K125C-S224C and K169C-Q194C, were labeled with Cy3/Cy5 c5 maleimide and then reconstituted into liposomes containing 30% cholesterol (w/w). Zn^{2+} was added to both sides of liposomes, and flux assays were started by adding 0.45 μM K^{+} ionophore valinomycin (Val) at 4 min time point (marked by black arrow), then 50 μM arachidonic acid (AA) was added at the 19 min time point, as indicated by blue and magenta arrows. Proton ionophore CCCP was used as a positive control, which led to maximum ACMA fluorescence quenching. All data were presented as mean±s.e, n=3.

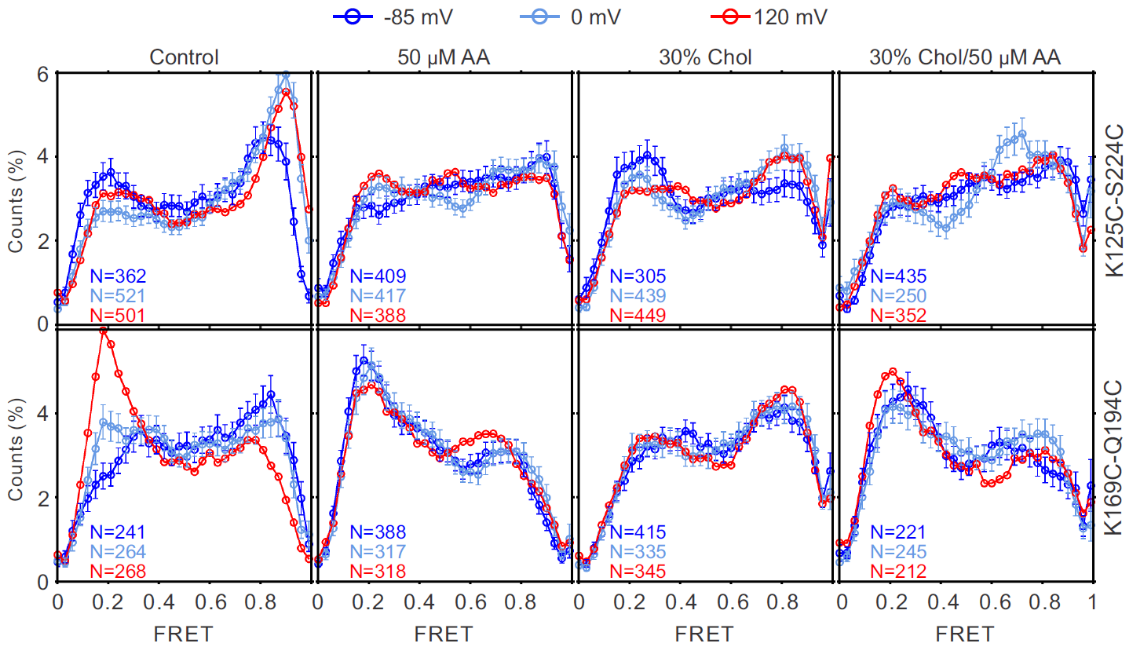

Fig S2. Arachidonic acid shifts conformational distributions of the hHv1 S4 segment.

Raw histograms were generated from smFRET data collected at K125C-S224C and K169C-Q194C labeling sites under -85, 0 and 120 mV. The bin size is 0.03, with trace number N included in each panel.

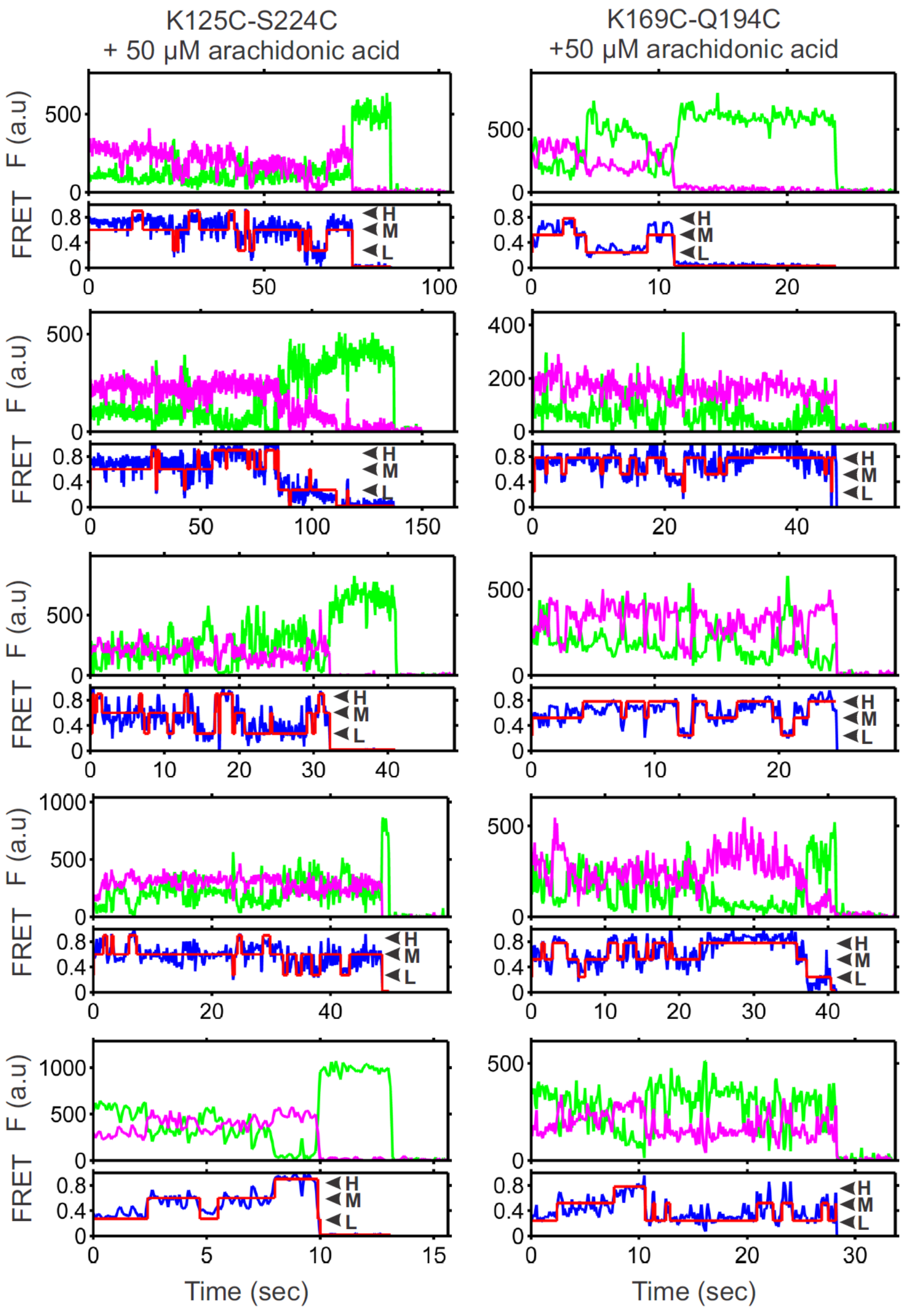

Fig S3. Representative smFRET traces exhibiting transitions among 3 FRET states

The smFRET data were collected at K125C-S224C and K169C-Q194C sites under -85 mV, with 50 μM arachidonic acid. The purple and green lines were donor and acceptor intensities, and the blue and red lines were real and idealized FRET. Low (L), medium (M), and high (H) FRET states in each trace were marked by arrows.
